# Nisin Damages the Septal Membrane and Triggers DNA Condensation in Methicillin-Resistant *Staphylococcus aureus*

**DOI:** 10.3389/fmicb.2020.01007

**Published:** 2020-06-03

**Authors:** Camilla Jensen, Heng Li, Martin Vestergaard, Anders Dalsgaard, Dorte Frees, Jørgen J. Leisner

**Affiliations:** ^1^Department of Veterinary and Animal Sciences, Faculty of Health and Medical Sciences, University of Copenhagen, Frederiksberg, Denmark; ^2^School of Chemical and Biomedical Engineering, Nanyang Technological University, Singapore, Singapore

**Keywords:** food preservative, TEM, SR-SIM, membrane depolarization, bacteriocin, antimicrobial resistance, time-killing, *Staphylococcus aureus*

## Abstract

Nisin is applied as a food preservative in processed foods and has the potential to be used synergistically with antibiotics for treatment of patients infected by antibiotic-resistant bacteria, such as methicillin-resistant *Staphylococcus aureus*. The present study explores the antimicrobial effect of nisin on *S. aureus* viability and membrane integrity and, for the first time, used super-resolution microscopy to study morphological changes induced in *S. aureus* cells exposed to nisin. The exposure of *S. aureus* to nisin caused membrane depolarization and rapid killing. Super-resolution structured-illumination microscopy and transmission electron microscopy confirmed that nisin damages the cellular membrane and causes lysis of cells. Strikingly, condensation of chromosomal DNA was observed in all cells exposed to nisin, a phenotype not previously reported for this compound. Moreover, cells exposed to nisin were significantly smaller than non-exposed cells indicating the emergence of cell shrinkage. The strong association of DNA condensation with nisin exposure indicates that nisin interferes with chromosome replication or segregation in *S. aureus*.

## Introduction

The commensal bacterium *Staphylococcus aureus* colonizes the nasal cavity of about one third of the human population and is a leading cause of bacterial infections with disease manifestations ranging from superficial skin infections to life-threatening invasive disease (van Belkum, 2006). Historically, β-lactam antibiotics have been the preferred choice for the treatment of staphylococcal infections. Effective treatment is, however, hampered by the rapid spread of methicillin-resistant *S. aureus* (MRSA) that have acquired resistance to virtually all members of the β-lactam class of antibiotics (Vestergaard et al., 2019). The emergence of community-acquired MRSA (CA-MRSA), such as the USA300 lineage, has further increased the global burden of *S. aureus* infections (Thurlow et al., 2012; Bal et al., 2016). Central to the pathogenicity of *S. aureus* are numerous secreted exotoxins, including the staphylococcal enterotoxins that are the causative agent of staphylococcal food poisoning, one of the most common causes of foodborne disease (Kadariya et al., 2014).

Nisin is an antibacterial peptide, classified as a class I bacteriocin that is active against a wide range of Gram-positive bacteria (Bierbaum and Sahl, 2009). For decades, nisin has been applied as a food preservative in processed foods, such as cheese, canned foods, semipreserved meats, and chocolate milk; occasionally with the aim to control the growth of *S. aureus* (Delves-Broughton, 1990; Felicio et al., 2015). More recently, the application of nisin has advanced beyond its role as a food bio-preservative. It has been shown to work synergistically with conventional therapeutic drugs against MRSA and as an agent to control biofilm formation by *S. aureus* and other pathogens (Brumfitt et al., 2002; Dosler and Gerceker, 2011; Field et al., 2016; Shin et al., 2016; Mathur et al., 2018).

Nisin targets the lipid II precursor that is the building block of peptidoglycan, the major constituent of the bacterial cell wall (Breukink et al., 1999; Breukink and de Kruijff, 2006). Additionally, nisin inserts into the cytoplasmic membrane resulting in pore formation and a lethal loss of membrane potential (Ruhr and Sahl, 1985; Wiedemann et al., 2001). Nisin-induced changes in *Bacillus* morphology suggest that membrane permeabilization by nisin is followed by accelerated cell division, cell envelope inhibition, and aberrant cell morphogenesis (Hyde et al., 2006). Membrane disruption and cell death has been correlated with the formation of nisin-lipid II aggregates in *B. subtilis* (Scherer et al., 2015). A study by Prince et al. (2016), however, suggests that cell death might be initiated by nisin-lipid II interactions but is driven by a membrane defect caused by alternative mechanisms. Hence, the downstream effects of nisin binding to lipid II leading to cell death appear to be complex. Notably, lipid II is the target of novel antibiotics, such as daptomycin and teixobactin, and for these compounds, the killing mechanism is not entirely understood (Muller et al., 2016; Oster et al., 2018).

To extend our knowledge on the potential for using nisin to control growth of MRSA strains, we here compare the nisin MICs of 18 *S. aureus* strains representing both methicillin-sensitive *S. aureus* (MSSA) and MRSA isolates. We find very little variation in nisin MIC values for MSSA and MRSA strains, suggesting that the SSC*mec* resistance determinant harbored by MRSA strains does not change susceptibility to nisin. Exposure of CA-MRSA cells to nisin first resulted in rapid killing; however, after 4 h, the MRSA strain was capable of regrowth. To gain a better understanding of the rapid bactericidal activity of nisin in *S. aureus*, we used super-resolution structured-illumination microscopy (SR-SIM) and transmission electron microscopy (TEM) to study the morphological changes induced by nisin in a CA-MRSA USA300 model strain. Interestingly, exposure of the MRSA cells to nisin for 30 min resulted in distinct morphological changes, including cell shrinkage, DNA condensation, and cell lysis.

## Materials and Methods

### Strains and Culture Conditions

Table 1 lists the *S. aureus* strains used in the experiments and their sources. Bacterial cultures were stored at -80°C in Brain Heart Infusion (BHI; Oxoid CM1135) broth containing 30% glycerol. Strains were cultured in Tryptic Soy Broth (TSB; Oxoid CM0129) at 37°C with aeration at 180 rpm for 18–24 h.

**TABLE 1:** The MIC of nisin in *S. aureus* strains.

**Strain**	**CC/MLS type**	**MIC of nisin (μg ml^–1^)**	**Source**	**References**
***S. aureus* (Methicillin-Resistant)**				
USA300 JE2	CC398/ST398	6.4	USA300, CA-MRSA strain	Fey et al., 2013
R1	CC398/ST398	12.8	Retail chicken	Tang et al., 2017
R2	CC398/ST398	12.8	Retail pork	Tang et al., 2017
R6	CC9/ST9	12.8	Retail pork	Tang et al., 2017
R11	CC398/ST398	12.8	Retail pork	Tang et al., 2017
R18	CC8/ST8	12.8	Retail pork	Tang et al., 2017
COL	CC8/ST250	12.8	Hospital-acquired	Dyke et al., 1966
***S. aureus* (Methicillin-Sensitive)**				
A1	CC101/ST101	12.8	Pasta salad	Li et al., 2019a
A5	CC779/ST779	12.8	Pasta salad	Li et al., 2019a
SS12	CC20/ST1281	12.8	Sushi	Li et al., 2019a, b
SS13	CC398/ST398	12.8	Sushi	Li et al., 2019a, b
SS60	CC45/ST45	12.8	Sushi	Li et al., 2019a, b
SA564	CC5/ST5	12.8	Clinical isolate	Somerville et al., 2002
USA300 JE2 △mecA	CC398/ST398	6.4	Methicillin sensitive	Fey et al., 2013
ATCC29213	CC5/ST5	12.8	Quality control strain	Soni et al., 2015
ATCC25923	CC30/ST243	12.8	Quality control strain	Treangen et al., 2014

### Minimum Inhibitory Concentration Analysis

Nisin A from *Lactococcus lactis* (2.5% in balance sodium chloride and denatured milk solids) was purchased from Sigma-Aldrich (N5764). Before each experiment, a fresh stock solution of nisin was prepared in 0.02 M HCl with a final concentration of 102.4 μg ml^–1^. Minimal inhibitory concentration (MIC) was determined following the Clinical and Laboratory Standards Institute 2017 guidelines^1^ in a 96-well format. Overnight cultures of *S. aureus* were diluted in physiological saline (0.9% NaCl) to reach turbidity of 0.5 McFarland (corresponding to ∼10^8^ CFU ml^–1^; Sensititre^®^ nephelometer and the Sensititre^®^ McFarland Standard). The bacterial suspensions were adjusted to 5 × 10^5^ CFU ml^–1^ in Mueller–Hinton broth (MH; Oxoid CM0405) in wells containing standard twofold dilutions of nisin in a final volume of 100 μl. The plates were incubated for 18–24 h with shaking (300 rpm) at 37°C. All experiments were performed in triplicate. MIC was defined as the concentration of nisin at which growth was inhibited.

### Time-Kill Curves

An overnight culture of *S. aureus* USA300 JE2 was diluted in physiological saline (0.9% NaCl) to reach turbidity of 0.5 McFarland as described before. Bacterial suspensions were adjusted to ∼5 × 10^6^ CFU ml^–1^ in Mueller–Hinton broth containing 0, 3.2, 6.4, 12.8, or 25.6 μg ml^–1^ of pure nisin in a final volume of 100 ml and incubated with aeration (180 rpm) at 37°C. Colony counts were determined by plating serial ten-fold dilutions on TSA every hour during the first 4 h and then after 12 and 24 h. Experiments were performed in duplicate. MIC determination of overnight cultures was performed for one of the duplicate experiments in order to determine susceptibility after prolonged exposure. Data analysis was computed in GraphPad Prism 5. Statistical significance was calculated using Student’s *t-*test. A value of *p* < 0.05 was considered significant.

### Assessment of Membrane Potential and Integrity Using Flow Cytometry

The membrane potential was assessed using a flow cytometry assay based on the BacLight Bacterial Membrane Potential Kit (Invitrogen, Thermo Fischer Scientific). 2 μl of cell suspension of stationary phase cultures of *S. aureus* USA300 JE2 were inoculated into six Falcon round-bottom tubes (14 ml, Corning), each containing 2 ml fresh TSB medium and grown to an OD_600_ of 0.2 (∼2 × 10^8^ CFU ml^–1^) at 37°C with shaking (180 rpm). At OD_600_ of 0.2, nisin (3.2, 6.4, 12.8, or 25.6 μg ml^–1^) was added to the tubes and incubated for 5 min. After 5 min of incubation with antimicrobials, 15 μl culture was transferred to 975 μl filtered phosphate-buffered saline. To each cell solution, 10 μl of fluorescent membrane potential indicator dye, DiOC_2_(3), was added, and cells were stained for 5 min at room temperature. The assay was verified by depolarizing the membrane with the protonophore carbonyl cyanide 3-chlorophenylhydrazone (CCCP) at a final concentration of 5 μM. Data was recorded on a BD Biosciences Accuri C6 flow cytometer (Becton, Dickinson and Company) with emission filters suitable for detecting red and green fluorescence. Settings on the flow cytometer were as follows: 25,000 recorded events at an FSC threshold of 15,000 and medium flow rate. Gating of stained cell population and analysis of flow cytometry data were performed in CFlow^®^ (BD Accuri). As an indicator of membrane potential, the ratio of red to green fluorescence intensity was calculated. Assessment of the membrane potential was performed with three biological replicates.

Membrane integrity was assessed using propodium iodide (PI). 2 μl of stationary USA300 JE2 cultures were transferred to six Falcon round-bottom tubes (14 ml, Corning), each containing 2 ml fresh TSB medium. Cultures were grown to an OD_600_ of 0.2 (∼2 × 10^8^ CFU ml^–1^) at 37°C with shaking (180 rpm). At OD_600_ of 0.2, CCCP (final conc.: 5 μM) and nisin (3.2, 6.4, 12.8, or 25.6 μg ml^–1^) were added to the tubes and incubated for 5 min. Then, 15 μl culture was transferred to 980 μl filtered phosphate-buffered saline and stained for 5 min with 5 μl of 0.1 mg ml^–1^ PI. After staining, red fluorescence levels in cells were recorded using a BD Biosciences Accuri C6 flow cytometer (Becton Dickinson). Settings on the flow cytometer were as follows: 25,000 recorded events at an FSC threshold of 15,000 and medium flow rate. Gating of stained cell population and analysis of flow cytometry data were performed in CFlow^®^ (BD Accuri). Assessment of the membrane integrity was performed with three biological replicates. The data were analyzed using GraphPad Prism 8 (GraphPad Software Inc., La Jolla, CA, United States) using one-way analysis of variance with a post-hoc analysis of Dunnett’s multiple comparison tests, where *P < 0.05, **P < 0.01, and ***P < 0.001.

### Super-Resolution Structured Illumination Microscopy (SR-SIM)

Prior to SR-SIM, a culture of *S. aureus* USA300 JE2 was grown in 50 ml of TSB at 37°C with a starting OD_600_ of 0.02. At OD_600_ ∼0.2 (∼2 × 10^8^ CFU ml^–1^), the culture was divided into three cultures of 10 ml; one grown in the absence of nisin, one supplemented with 3.2 μg ml^–1^ nisin, and one supplemented with 6.4 μg ml^–1^ nisin. The three cultures were incubated for 30 min at 37°C. Then, 1 ml samples of the cultures were stained for 5 min at room temperature with the membrane dye Nile Red, the cell wall dye WGA-488, and the DNA dye Hoechst 33342 (Supplementary Table S1). Samples were placed on an agarose pad (1.2% in PBS) and visualized by SR-SIM. Images were acquired using an Elyra PS.1 microscope (Zeiss) with a Plan-Apochromat 63×/1.4 oil DIC M27 objective and a Pco.edge 5.5 camera with five grid rotations. Images were reconstructed using ZEN software (black edition, 2012, version 8.1.0.484) based on a structured illumination algorithm, using synthetic, channel-specific optical transfer functions and noise filter settings ranging from -6 to -8. Laser specifications are shown in Supplementary Table S1. SR-SIM was performed at the Core Facility for Integrated Microscopy (CFIM), Faculty of Health and Medical Sciences, University of Copenhagen.

### Estimation of Cell Size

The volume of 200 cells were determined as described by Monteiro et al. (2015) with slight modification. Briefly, an ellipse was fitted to the edge of the outer cell wall, and the lengths of the minor and major axes were acquired. The shape of the cells were assumed to be that of a prolate spheroid; hence, the volume was estimated using the equation V = 4/3π*ab*^2^, where *a* and *b* correspond to the length of the major and minor axes, respectively. Ellipse fitting and measurements were performed in Image J.

### Transmission Electron Microscope (TEM)

Prior to TEM, *S. aureus* cultures were grown as described for SR-SIM. Cells were harvested at 8,000 × *g*, and pellets were suspended in fixation solution [2.5% glutaraldehyde in 0.1 M cacodylate buffer (pH 7.4)] and incubated overnight at 4°C. The fixed cells were treated with 2% osmium tetroxide, followed by 0.25% uranyl acetate for contrast enhancement. The pellets were dehydrated in increasing concentrations of ethanol, followed by pure propylene oxide. Cells were finally embedded in Epon resin, and thin sections were stained with lead citrate. Images were acquired with a Philips CM100 BioTWIN transmission electron microscope fitted with an Olympus Veleta camera with a resolution of 2,048 by 2,048 pixels. Sample preparation and microscopy were performed at the CFIM.

## Results and Discussion

### MIC of Nisin Toward *S. aureus*

The majority of the included *S. aureus* strains originated from a range of food products, including retail pork and chicken products, pasta salad, and sushi. The strains represent a range of different clonal complex types (Li et al., 2019a), and they represented both methicillin-sensitive and -resistant isolates as specified in Table 1. In the broth dilution assays, all tested *S. aureus* isolates exhibited similar nisin MIC values (6.4–12.8 μg ml^–1^; Table 1) as a two-fold deviation in MIC values is considered non-significant according to the International Organization for Standardization (ISO-20776-1, 2006). Hence, our results show that methicillin-resistant and -sensitive strains have similar nisin MICs, indicating that the *SCC*Mec cassette, encoding the *mecA* gene conferring methicillin resistance to MRSA strains, does not impact susceptibility to nisin. In support of this conclusion, the nisin MIC did not change by deletion of the *mecA* gene in the JE2 strain background (Table 1). JE2 represents the fast-spreading and highly virulent community-acquired MRSA USA300 clone, and accordingly, JE2 has become a preferred MRSA model strain (Fey et al., 2013). For this reason, we used the JE2 strain to further characterize the response of *S. aureus* to nisin exposure.

### Effect of Nisin on Viability of *S. aureus*

**FIGURE 1:**
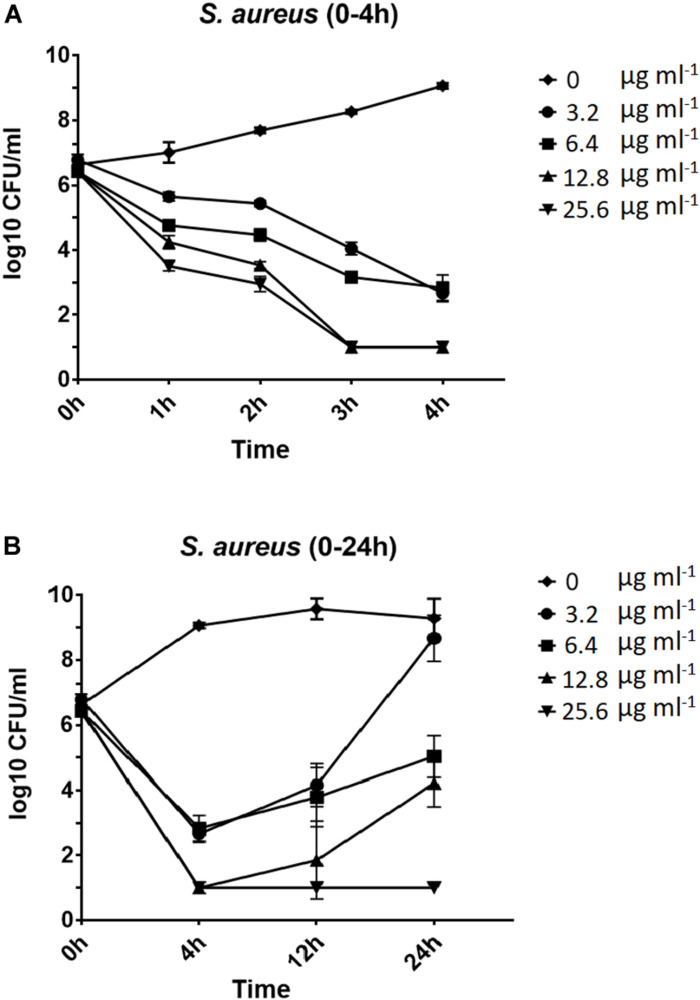
Time-kill curves. Overnight cultures of *S. aureus* USA300 JE2 adjusted to ∼5 × 10^6^ CFU ml^−1^ was exposed to 0, 3.2, 6.4, 12.8, or 25.6 μg ml^−1^ nisin. Viability was assessed by determining CFU ml^−1^ every hour during the first 4 h after nisin exposure **(A)** and following 4, 12, and 24 h after nisin exposure **(B)**. Experiments were performed in duplicate.

Next, time-kill assays were performed to assess the viability of *S. aureus* cells exposed to nisin for 1–24 h. For all tested concentrations of nisin (3.2–25.6 μg ml^–1^), the viable count decreased during the first 4 h in a time- and concentration-dependent manner (Figure 1A). In particular, the viable cell count was significantly reduced (*P* < 0.01 for 3.2 and 6.4 μg ml^–1^; *P* < 0.0001 for 12.8 and 25.6 μg ml^–1^) within the first hour of exposure to nisin (Figure 1A), demonstrating that nisin has bactericidal activity against *S. aureus*. Interestingly, the viable count started to increase in cultures exposed to 3.2–12.8 μg ml^–1^ nisin for more than 4 h (Figure 1B), and after 24 h, cells exposed to 3.2 μg ml^–1^ nisin reached almost the same final yield as non-exposed cells (Figure 1B). To address, if the regrowth phenotype observed in cultures exposed to nisin for >4 h was due to the selection of nisin-resistant mutants, we examined the MIC of colonies recovered after 24 h exposure to 3.2 μg ml^–1^ nisin. However, as the nisin MIC of all tested colonies remain unaltered (data not shown), the ability of *S. aureus* to resume growth after 4 h of nisin exposure does not seem to be caused by stable mutations. Instead, a minor fraction of the cell population seems capable of adapting to nisin at the post-genetic level. Consistent with our results, Brumfitt et al. (2002) found that nisin reduced the viable count of 20 MRSA strains drastically within the first 2 h of the experiment, however, following the initial killing, 11 of the MRSA strains were capable of regrowth while remaining fully sensitive to nisin.

### Membrane Depolarization of *S. aureus* Cells Exposed to Nisin

**FIGURE 2:**
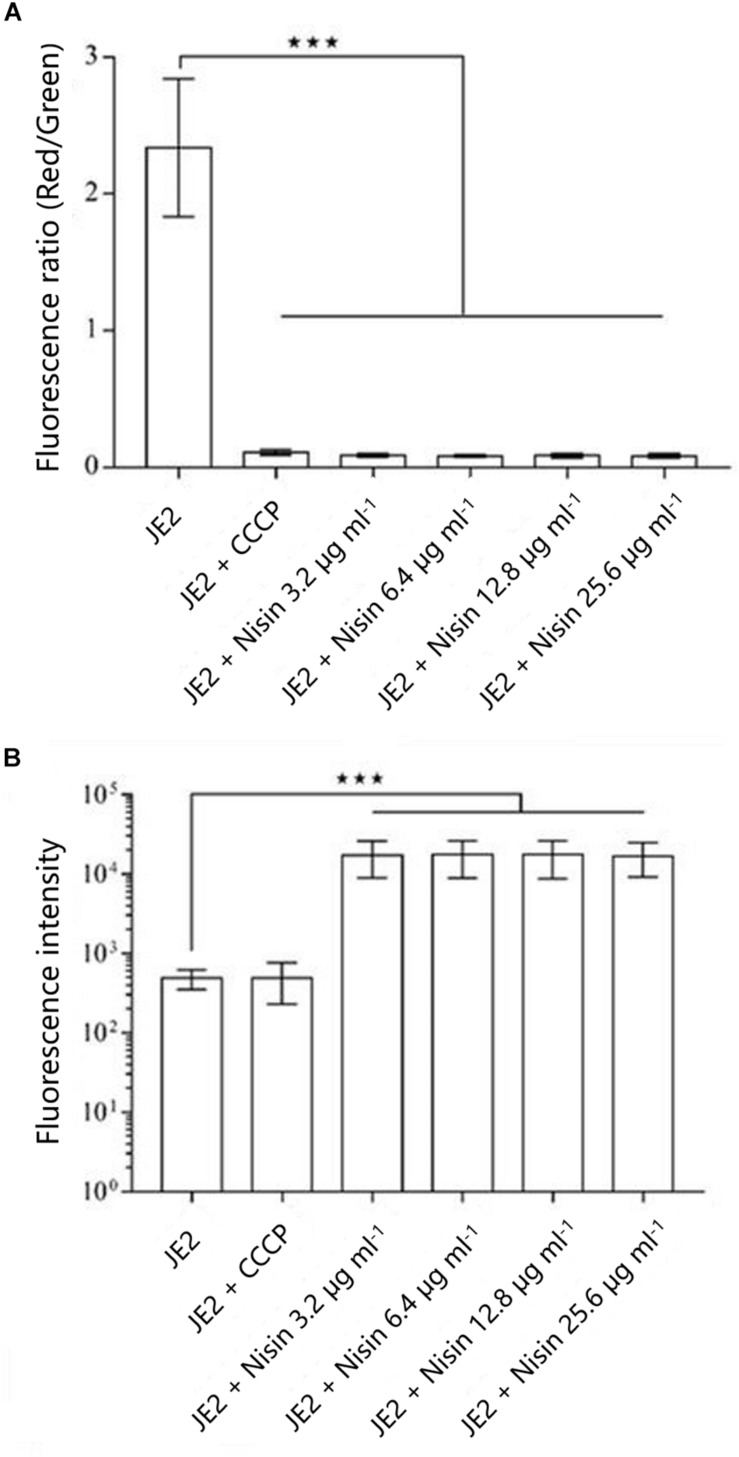
**(A)** Nisin reduces membrane potential in *S. aureus*. The membrane potential was assayed using the fluorescent dye DiOC_2_(3), which exhibits green fluorescence in bacterial cells and shifts toward red fluorescence when the dye molecules self-associate at higher cytosolic concentrations at higher membrane potentials. The red/green ratio is a proxy for the membrane potential. The protonophore CCCP was included as a control for membrane depolarization. Each group displays the average of three measurements, and the error bars display 95% confidence intervals. *P < 0.05; **P < 0.01; ***P < 0.001. **(B)** Nisin compromises the cell membrane of *S. aureus*. Membrane integrity was assayed using propidium iodide that stains cells with compromised membranes. Each group displays the average of three measurements and the error bars display 95% confidence intervals. *P < 0.05; **P < 0.01; ***P < 0.001.

*S. aureus* USA300 JE2 treated with nisin (3.2–25.6 μg ml^–1^) resulted in membrane depolarization (Figure 2A), which is likely a result of pore formation in membranes following nisin treatment as the uptake of the PI was also increased (Figure 2B). In contrast, the protonophore CCCP resulted in membrane depolarization without increased PI uptake (Figures 2A,B) as previously described for *S. aureus* (Parsons et al., 2012) and *Bacillus subtilis* (McAuley et al., 2018). Our findings support previous studies (Parsons et al., 2012; McAuley et al., 2018), indicating that nisin depolarizes cells by creating pores in the membrane rather than acting as a protonophore.

### Nisin Damages the Cellular Membrane and Triggers DNA Condensation of USA300

The small size of the staphylococcal cells has, until recently, hampered a detailed analysis of live *S. aureus* exposed to antibiotics. Recent advances in super-resolution structured-illumination microscopy (SR-SIM) in combination with novel staining techniques have, however, shown great potential for expanding our knowledge on how antibiotics impact vital cellular processes, such as cell wall synthesis and cell division in *S. aureus* (Monteiro et al., 2018; Jensen et al., 2019). To improve our understanding of why exposure to nisin leads to rapid killing, we here used SR-SIM to study the morphological changes occurring in *S. aureus* cells exposed to 6.4 μg ml^–1^ nisin for 30 min. Prior to SR-SIM imaging, cells were stained with fluorescent dyes that specifically label different cellular structures. The membrane was stained red using the Nile Red membrane dye, the DNA was stained blue using the Hoechst 33342 DNA dye, and the peripheral wall was stained green with WGA-488 that specifically binds to N-acetylglucosaminyl and sialic acid residues on cell surfaces. As WGA-488 is too big to penetrate into the septum, it selectively labels the peripheral wall.

**FIGURE 3:**
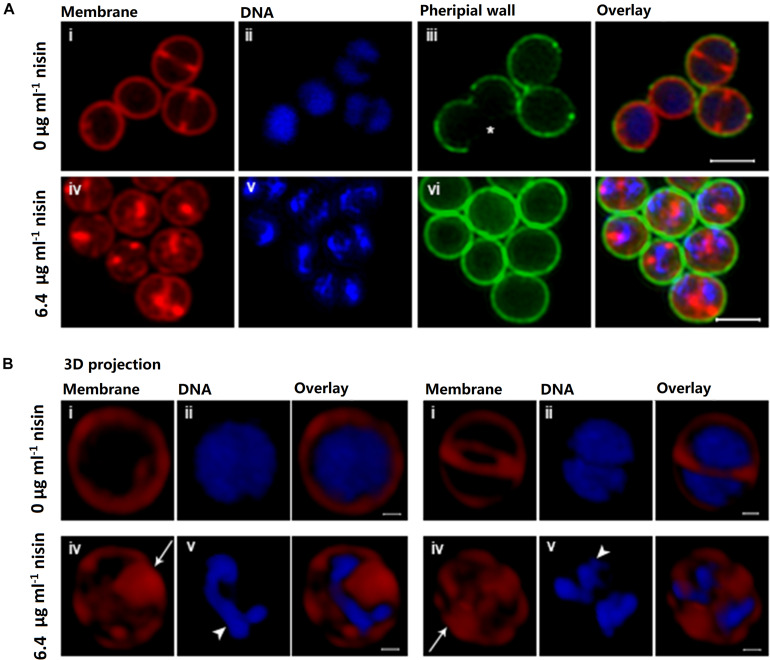
Nisin triggers severe membrane damage and DNA condensation in *S aureus*. SR-SIM images of USA300 JE2 wild-type cells grown in the absence (upper panels in **A,B**) or presence of nisin for 30 min (lower panels in **A,B**). Prior to imaging, membranes were stained red with Nile Red, the peripheral cell wall was stained green with WGA-488, and DNA was stained blue as depicted in the figure. In **(A)**, SR-SIM images of multiple cells are displayed in 2-D (scale bar, 1 μm), and 3-D structures (transparency projection) of enlarged cells (scale bar, 0.2 μm) are shown in **(B)**. Cells exposed to nisin show bulging of membranes (white arrows) and condensation of nucleoid DNA (white arrowhead). Daughter cells that have separated following WGA labeling display a characteristic labeling pattern with parts of septal origin being unstained by WGA (asterisk). Daughter cell splitting is not observed in cells exposed to nisin.

*S. aureus* cells grown in the absence of nisin exhibited the characteristic spherical shape surrounded by a smooth and regular membrane (Figures 3A-i,B-i) and with the DNA uniformly distributed in the entire cytoplasm of non-dividing cells and on each side of the division septum in dividing cells (Figures 3A-ii,B-ii). In contrast, cells exposed to nisin displayed striking differences as characterized by bulging and invaginations in the membrane (Figures 3A-iv,B-iv); in particular, the septal Nile Red signal appears bulky and non-uniform (Figure 3B-iv, white arrows). This suggests that nisin triggers membrane damage that results in disposure of unstructured membranous material at the septal site. Most strikingly, however, the DNA in cells exposed to nisin appeared condensed and fragmented (Figure 3A-v and white arrowheads in Figure 3B-v) indicating that exposure of *S. aureus* to nisin, in addition to conferring loss of membrane integrity, confers changes in the structure of chromosomal DNA, most likely by an indirect mechanism. Moreover, nisin seems to impair the splitting of *S. aureus* daughter cells as none of the nisin-treated cells showed the staining pattern that characterizes daughter cells having separated following WGA staining (marked with an asterisk in Figure 3A: in newly separated cells, cell wall of septal origin is non-stained as WGA cannot penetrate and label the septal cell wall; Jensen et al., 2019). Cells exposed to nisin were also significantly smaller than non-exposed cells (*V* = 0.68 ± 0.2 μm^3^ as compared to *V* = 0.87 ± 0.2 μm^3^, *P* < 0.0001), indicating that exposure to nisin induces cell shrinkage. Finally, nisin seems to impair the splitting of *S. aureus* daughter cells as none of the nisin-treated cells showed the characteristic staining pattern, indicating that cells have separated following WGA staining (Figure 3A). Similar results were obtained when cells were exposed to 3.2 μg ml^–1^ nisin for 30 min.

**FIGURE 4:**
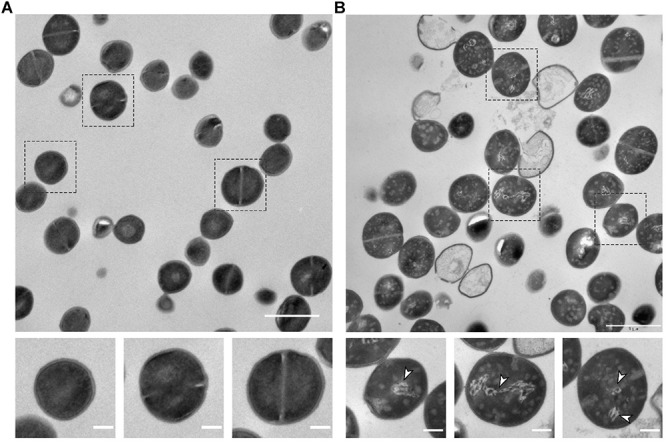
TEM confirms nisin-induced DNA alteration and lysis. TEM images of USA300 JE2 wild-type cells grown to mid-exponential phase at 37°C in the absence **(A)** or presence of 6.4 μg ml^– 1^ nisin for 30 min. **(B)** Boxes in the upper panels are enlarged in the lower panels. White arrowheads point to electron light regions corresponding to condensed and fragmented DNA in the lower panels. Scale bar, 1 μm (upper panel) and 0.2 μm (lower panel).

The morphological changes induced by nisin were additionally investigated using transmission electron microscopy (TEM). Figure 4 shows representative electron micrographs of untreated cells (Figure 4A) and cells exposed to 6.4 μg ml^–1^ (Figure 4B). Interestingly, the TEM images confirmed that nisin induces profound alterations in the DNA structure as condensed electron-light regions (Figure 4B, arrowheads) that most likely correspond to condensed and fragmented DNA that were clearly visible only in cells exposed to nisin. In contrast, the DNA is not clearly distinguishable in the cytoplasm of non-treated cells (Figure 4A). Interestingly, in the presence of nisin, approximately 14% of cells appeared as lysed ghost cells in the TEM images (Figure 4), demonstrating that exposure to nisin results in rapid cell lysis. Nucleoid condensation was previously observed in *S. aureus* cells with impaired chromosome segregation, and in *S. aureus* cells exposed to the fluoroquinolone antibiotic nalidixic acid that induces DNA damage by targeting the DNA gyrase (Pereira et al., 2013; Veiga and Mariana, 2017). In these studies, DNA condensation was also accompanied by membrane bulges, and interestingly, the alterations in the membrane were proposed to be a consequence rather than a cause of DNA condensation. Therefore, the strong association of DNA condensation with nisin exposure indicates that nisin interferes with chromosome replication or segregation in *S. aureus*.

## Conclusion

We here show that exposure to nisin causes membrane depolarization and rapid killing of the JE2 CA-MRSA model strain belonging to the fast spreading and highly virulent USA300 clone. Super-resolution microscopy and transmission electron microscopy confirmed that nisin damages the cellular membrane and causes lysis of JE2 cells. Strikingly, condensation of chromosomal DNA was observed in all JE2 cells exposed to nisin, a phenotype not previously reported for this compound. The strong association of DNA condensation with nisin exposure indicates that chromosome replication or segregation is impeded in *S. aureus* cells exposed to nisin. Therefore, DNA damage might be a crucial component in the killing mechanism of nisin in *S. aureus*, and future studies should be directed to test this phenomenon in *S. aureus* strains of different origins and belonging to different clonal complexes.

## Data Availability Statement

The datasets generated for this study are available on request to the corresponding author.

## Author Contributions

HL, CJ, DF, and JL designed the study. HL, CJ, and MV performed the experiments. HL, CJ, MV, AD, DF, and JL analyzed the data and wrote the manuscript.

## Conflict of Interest

The authors declare that the research was conducted in the absence of any commercial or financial relationships that could be construed as a potential conflict of interest.

## Footnotes

1 https://clsi.org/
