# Supplementary material for: Nisin Damages the Septal Membrane and Triggers DNA Condensation in Methicillin-Resistant Staphylococcus aureus
Source: Front Microbiol. 2020 Jun 3;11:1007. doi: 10.3389/fmicb.2020.01007 (PMC7283504; doi:10.3389/fmicb.2020.01007)
Supplement: Supplementary file 1 [file Table_1.pdf]

Table S1: Staining and laser specifications used for SR-SIM

Staining	Concentration	Target	Laser	Laser Type	Laser power	Beam splitter	Grating
Nile Red	5 µg ml^{-1}	Membrane	561 nm	HR Diode – 100mW	8 % / 100 ms	BP 570-650 + LP 750	34 µm
HADA	1 µg ml^{-1}	DNA	405 nm	HR Diode – 50mW	50 % / 100 ms	BP 420-480 + LP 750	23 µm
WGA-488	1 µg ml^{-1}	Old PG	488 nm	HR Diode – 100mW	5 % / 100 ms	BP 495-575 + LP 750	28 µm
